# Correlation Analysis between *Helicobacter pylori* Infection Status and Tumor Clinical Pathology as Well as Prognosis of Gastric Cancer Patients

**Published:** 2018-10

**Authors:** Jing WANG, Xiaofeng LIU

**Affiliations:** 1. Clinical Laboratory, Weihaiwei People’s Hospital, Weihai 264200, China; 2. Clinical Laboratory, Pingdu People’s Hospital, Pingdu 266700, China

**Keywords:** Gastric cancer, *Helicobacter pylori*, Clinical pathology, Prognosis, Correlation analysis

## Abstract

**Background:**

We aimed to investigate the correlation between *Helicobacter pylori* infection and the development and prognosis of gastric cancer.

**Methods:**

Retrospective analysis was used to collect 120 paraffin-embedded specimens and 42 paracancerous specimens of gastric cancer patients archived in Department of Pathology, Weihaiwei People’s Hospital from 2010–2012. All patients with gastric cancer were followed for 5 years. Real-time fluorescent quantitative PCR was used to detect the relative *H. pylori* infection in gastric cancer tissues and paracancerous tissues. The relationship between clinicopathological parameters and *H. pylori* relative infection was analyzed. Kaplan-Meier was used for survival analysis.

**Results:**

The relative amount of *H. pylori* infection in gastric cancer tissues was significantly higher than that in paracancerous normal tissues (*P*<0.001). The relative *H. pylori* infection was related to tumor size, lymph node metastasis, clinical stage, and depth of invasion (*P*<0.05). The 1-, 3-, and 5-year survival rates of gastric cancer patients were negatively correlated with the relative *H. pylori* infection. The relative *H. pylori* infection, age, tumor size, lymph node metastasis, distant metastasis, clinical stage and depth of invasion were positively correlated with the prognosis of patients with gastric cancer.

**Conclusion:**

The relative *H. pylori* infection and clinical stage of patients could increase the risk of death in gastric cancer patients. *H. pylori* is one of the independent risk factors for the progression and prognosis of gastric cancer. It is also an index to evaluate the development process and prognosis of gastric cancer.

## Introduction

Gastric cancer is a common tumor worldwide. The number of newly-diagnosed patients is approximately 951,000 and the death toll is approximately 723,000 (1). The burden of gastric cancer in developing countries is heavier than in developed countries (2). The occurrence and development of gastric cancer is a complex process in which multiple factors, genes, and stages interact with each other. Smoking, smoked products, high dietary salt, high sugar, and other unhealthy lifestyles can increase the risk of gastric cancer (3). At present, the main method for the treatment of gastric cancer is still the comprehensive treatment based on surgery, but the local recurrence rate is still as high as 50%, postoperative 5-year survival rate is only 20%–50% (4, 5).

*Helicobacter pylori* infection is one of the most important factors involved in stomach canceration. The incidence of gastric cancer and peptic ulcers increases with the increase of *H. pylori* infection rate (6). *H. pylori* has been included as the first type of carcinogen by the International Agency for Research on Cancer (7). At the same time, most scholars have recognized *H. pylori*’s role in screening for gastric cancer and its role in the development and prognosis of gastric cancer (8–10). At present, the specific pathogenesis of gastric cancer has not yet been clarified. There is no definitive conclusion on *H. pylori* infection and tumor development and prognosis. It has been reported that the occurrence of gastric cancer is closely related to *H. pylori* infection, and *H. pylori* can cause gastric mucosal damage by inducing inflammatory responses through the regulation of relevant signaling pathways, thereby affecting the prognosis of gastric cancer patients (11–13).

The purpose of this study was to investigate the relationship between *H. pylori* infection and the progression and prognosis of gastric cancer in patients with gastric cancer, and to guide the diagnosis, treatment and prognosis of gastric cancer.

## Materials and Methods

### Sample collection

A retrospective method was used to collect 120 paraffin-embedded specimens and 42 paraneoplastic tissues from patients with gastric cancer who were first surgically resected from October 2010 to October 2012 in Weihaiwei People’s Hospital.

This study was approved by the Ethics Committee of Weihaiwei People’s Hospital, Weihai, China. All patients or their families have signed the informed consent.

The average age of 120 patients was 54.24±16.48 yr, including 79 males and 41 females. The adjacent tissue was a non-cancer tissue with a margin of 5 cm from the edge of the lesion. Specimens were fixed with formaldehyde. All patients had no chemotherapy or radiotherapy before surgery. All cases were histopathologically confirmed as gastric cancer and had not received anti- *H. pylori* treatment. All patients were excluded from other parts and tissues of primary and malignant tumors, heart, liver, and renal insufficiency, and gastric surgery was not performed within six months before admission. All subjects and their families signed informed consent.

### Main reagents and instruments

Qiagen 56404 QIAamp DNA FFPE Tissue Kit Paraffin Tissue DNA Extraction Kit and Qiagen 204054 QuantiFast SYBR Green PCR Kit were purchased from QIAGEN; Nanodrop 2000 UV spectrophotometer was purchased from Thermo Scientific, USA; Ro-tor-Gene Q-PCR instrument Purchased from QIAGEN.

### DNA extraction

The paraffin tissues were sectioned into 20 pieces with a thickness of 5 μm and loaded into a sterile EP tube. Qiagen 56404 QIAamp DNA FFPE Tissue Kit was used to extract DNA from gastric cancer and paracancerous tissues. DNA purity and mass concentration were measured using a Nanodrop 2000 UV spectrophotometer.

### Primer design

This experiment was designed and synthesized by Sangon Biotech (Shanghai) Co., Ltd.. using the *H. pylori* marker gene HPYR1 and GAPDH as an internal reference gene. The primer sequences are showed in Table 1.

**Table 1:** HPYR1 primers and internal reference sequences

	***Upstream primers***	***Downstream primers***
HPYR1	5′-GAGCCCTCAAAGAACTGCAC-3′	5′-AATTGGACAGCACCTTCTGG-3′
GAPDH	5′-TCAACGACCACTTTGTCAAGCTCA-3′	5′-GCTGGTGGTCCAGGGGTCTTACT-3′

### Real-time PCR detection

The real-time PCR reaction system was prepared according to the instructions and a total of 25 μL was prepared: 12.5 μL of 2×Quantifast SYBR Green PCR Master Mix, 1 μL each of 10 μmol primers, and 3 μL of template DNA. Finally, RNase-free water was used to complete to 25 μL. Using the Ro-tor-Gene Q real-time fluorescence quantitative PCR instrument for PCR amplification, the reaction conditions were: 95°C 5 min, 95 °C 20 s, 60 °C 45 s, a total of 45 cycles. The PCR product was stored at 4°C. GAPDH was used as the internal control, and 2^−ΔCt^ method was used to analyze the relative *H. pylori* infection in the specimens. The average value of the experiment was repeated three times.

### Follow-up

The patients in this group were followed up by telephone and outpatient follow-up. All patients with gastric cancer were followed for 5 years. The relationship between the relative *H. pylori* infection and the clinicopathological features of gastric cancer was observed, and the relationship between the relative *H. pylori* infection and patient survival was analyzed.

### Statistical methods

SPSS21.0 statistical software package (Cabit Information Technology Co., Ltd.) was used for statistical analysis of data. Measurement data were analyzed by *t*-test, Kaplan-Meier survival analysis was used, Log Rank test was used to compare survival rates among different *H. pylori* infections, and Cox regression was used to analyze the relationship between relevant variables and clinical prognosis. *P*<0.05 was considered statistically significant.

## Results

### Relative infections of H. pylori in two groups

**Fig. 1:**
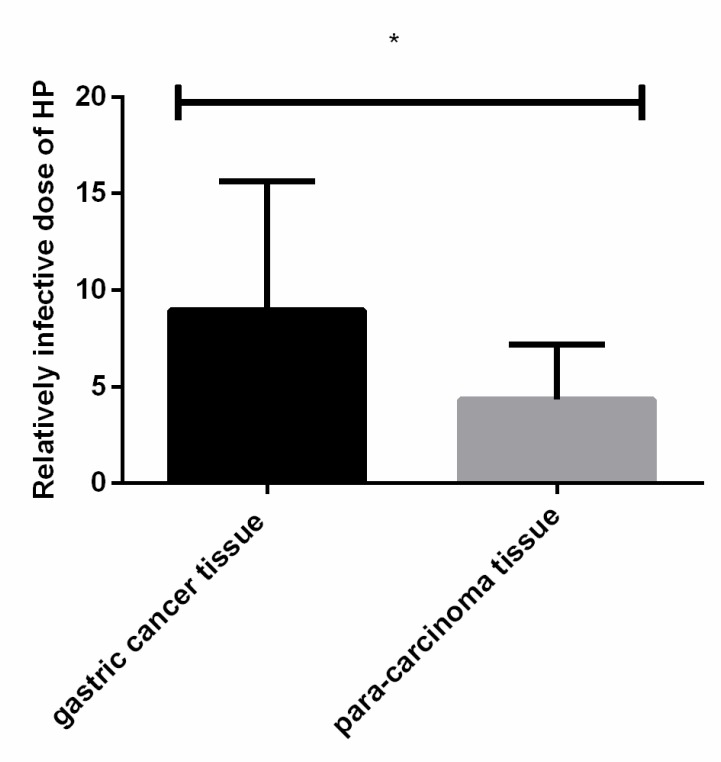
Relative *H. pylori* infection in both groups. Real-time fluorescence quantitative PCR results showed that the relative amount of *H. pylori* infection in gastric cancer tissue was significantly higher than that in paracancerous normal tissues, and the difference was statistically significant (*P*<0.001) Note: *<0.001 compared with paracancerous normal tissues

The relative amount of *H. pylori* infection in gastric cancer tissues (8.94±6.71) was significantly higher than that in paracancerous normal tissues (4.32±2.87). The difference was statistically significant (*P*<0.001) (Fig. 1 and Table 2).

**Table 2:** *H. pylori* relative infection

***Group***	***n***	**H. pylori *relative infection***	**t**	***P***
Gastric cancer tissue	120	8.94±6.71	4.319	< 0.001
Paracancerous tissue	42	4.32±2.87		

### Relationship between relative infections of H. pylori and clinicopathological features of gastric cancer

There was no significant correlation between *H. pylori* relative infection and age, gender, distant metastasis, and the difference was not statistically significant (*P*>0.05). The relative *H. pylori* infection was correlated with tumor size, lymph node metastasis, clinical stage, and depth of invasion. The difference was statistically significant (*P*<0.01) (Table 3).

**Table 3:** Relationship between relative infections of *H. pylori* and clinicopathological features of gastric cancer

***Variety***		***Number of cases (n=120)***	**H. pylori *relative infection***	**t**	***P***
Age					
	≥54	75	9.56±5.46	0.0	0.985
	<54	45	9.58±5.84	19	
Gender					
	Male	79	10.49±5.76	1.6	0.108
	Female	41	8.65±6.17	20	
Tumor size					
	≥5 cm	46	10.73±5.24	4.2	< 0.001
	<5 cm	74	7.12±4.11	04	
Lymph node metastasis										
	Yes	53	11.47±5.23	3.7	< 0.001
	No	67	7.89±5.12	68	
Distant metastasis										
	Yes	106	9.64±6.46	0.1	0.905
	No	14	9.43±3.47	19	
Clinical staging										
	I+II	55	7.82±6.15	2.5	0.013
	III+IV	65	10.46±5.28	30	
Infiltration depth										
	T1+T2	37	6.89±6.12	4.1	< 0.001
	T3+T4	83	11.42±5.16	89	

### The relationship between the survival rate of gastric cancer and the relative infection of H. pylori

**Fig. 2:**
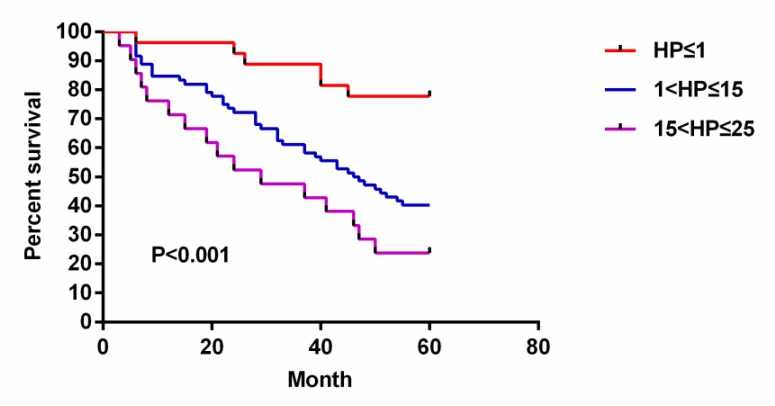
Relationship between gastric cancer survival rate and relative *H. pylori* infection. Kaplan-Meier survival analysis showed that the higher the relative *H. pylori* infection, the lower the survival rate of gastric cancer patients, and the 1-, 3-, and 5-year survival rates of gastric cancer patients were negatively correlated with the relative *H. pylori* infection. The 5-year survival rate was 77.78% in the *H. pylori*≤1 group, 40.28% in the 1<*H. pylori*≤15 group, and 23.81% in the 15<*H. pylori*≤25 group. The difference between the three groups was statistically significant (*P*<0.001)

The higher the relative *H. pylori* infection, the lower the survival rate of gastric cancer patients. The 1-year, 3-year and 5-year survival rates of gastric cancer patients were negatively correlated with the relative *H. pylori* infection (Fig. 2 and Table 4).

**Table 4:** The relationship between the survival rate of gastric cancer and the relative infection of *H. pylori*

***Relative infection***	***n***	***Survival rate[(n)%]***			***P***
		***1-year***	***3-year***	***5-year***	
*H. pylori*≤1	27	26 (96.30)	23 (85.19)	21 (77.78)	< 0.001
1 < *H. pylori*≤15	72	61 (84.72)	43 (59.72)	29 (40.28)	
15 < *H. pylori*≤25	21	15 (71.43)	10 (47.62)	5 (23.81)	

### Cox regression analysis of clinical pathological characteristics and relative H. pylori infection in gastric cancer

Cox regression univariate analysis showed that the prognosis of patients with gastric cancer was positively correlated with the relative *H. pylori* infection, age, tumor size, lymph node metastasis, distant metastasis, clinical stage, and depth of invasion. Multivariate analysis showed that the relative *H. pylori* infection and clinical stage of patients could increase the risk of death in gastric cancer patients, and it was also an independent risk factor for the development process and poor prognosis of gastric cancer (Table 5 and 6).

**Table 5:** Single factor analysis of prognosis of gastric cancer patients

***Group***	***Regression coefficients***	***Relative risk***	***P***
*H. pylori* relative infection	0.089	1.087	< 0.001
Age	0.031	1.034	0.016
Gender	0.021	0.652	0.421
Size of tumor	0.752	2.128	< 0.001
Lymph node metastasis	0.824	2.408	< 0.001
Distant metastasis	1.516	4.324	< 0.001
Clinical staging	1.171	2.956	< 0.001
Infiltration depth	0.957	2.649	< 0.001

**Table 6:** Multivariate analysis of prognosis of gastric cancer patients

***Group***	***Regression coefficients***	***Relative risk***	***P***
*H. pylori* relative infection	0.062	1.059	0.008
Age	0.038	1.039	0.055
Gender	0.084	1.043	0.856
Size of tumor	0.534	1.507	0.146
Lymph node metastasis	0.367	1.382	0.124
Distant metastasis	0.288	1.412	0.625
Clinical staging	0.851	2.456	0.014
Infiltration depth	0.052	1.125	0.885

## Discussion

*H. pylori* can secrete more antioxidative enzymes to prevent it from being killed by gastric neutrophils, and it can hydrolyze the specific protective layer produced by urea to resist the killing by gastric acid (14). A large number of reports have reported that persistent *H. pylori* infection may induce gastritis, further induce gastric mucosal cell damage, infiltrate inflammatory cells, and cause deterioration of gastric mucosal cells (15–17). *H. pylori* can cause gastric precancerous lesions, but also can cause oncogene mutations, leading to the occurrence of gastric cancer (18). Foreign research reports show that *H. pylori* infection is involved in the entire process of gastric cancer development (18, 19).

This study showed that the relative amount of *H. pylori* infection in gastric cancer tissue was higher than that in normal tissues adjacent to the cancer. This indicates that *H. pylori* can promote the occurrence of gastric cancer. The results of Wroblewski's study (20) are consistent with ours. He indicates that the prevalence of gastric cancer in *H. pylori* -infected patients is much higher than that in non-infected patients, and total gastrectomy for gastric cancer patients and eradication of *H. pylori* are beneficial to gastric cancer patients.

In this study, there was no significant correlation between the relative amount of *H. pylori* infection and age, gender, distant metastasis. The relative amount of *H. pylori* infection was correlated with tumor size, lymph node metastasis, clinical stage, and depth of infiltration, and the difference was statistically significant. The results of other studies (21, 22) are basically consistent with ours. *H. pylori* infection is associated with gastric cancer infiltration (22).

Most patients showed *H. pylori* positive in the T1 and T2 staging of gastric cancer, indicating that *H. pylori* is involved in the metastasis of gastric cancer. *H. pylori* infection was associated with lymph node metastasis (22). Most patients with gastric cancer showed positive *H. pylori* in the N0 and N1 stages. The higher the relative *H. pylori* infection, the lower the survival rate of gastric cancer patients. The 1-year, 3-year and 5-year survival rates of gastric cancer patients were negatively correlated with the relative *H. pylori* infection.

Cox regression univariate analysis showed that the prognosis of patients with gastric cancer was positively correlated with the relative *H. pylori* infection, age, tumor size, lymph node metastasis, distant metastasis, clinical stage, and depth of invasion. Multivariate analysis showed that the relative *H. pylori* infection and clinical stage of patients could increase the risk of death in gastric cancer patients, and it was also an independent risk factor for the development process and poor prognosis of gastric cancer. For the report of the relationship between the prognosis of gastric cancer and *H. pylori* infection, the results of the study are mixed. Patients with advanced *H. pylori* and metastases have higher sensitivity to chemotherapy than patients with *H. pylori*-negative gastric cancer, so the prognosis is better (23). *H. pylori* infection plays a protective role in the prognosis of gastric cancer (24). In contrast, gastric cancer patients not infected with *H. pylori* had a better prognosis (25). Relative *H. pylori* infection was an independent risk factor for poor prognosis (26). The reason for the difference in results may be due to differences in the treatment effect, age, and clinical stage of the study subjects, or it may be due to the fact that some patients had undergone *H. pylori* radical resection prior to surgery.

## Conclusion

*H. pylori* infection is related to the clinicopathological data of gastric cancer patients. *H. pylori* is one of the independent risk factors for the development and prognosis of gastric cancer, and it is also an index to evaluate the development process and prognosis of gastric cancer.

## Ethical considerations

Ethical issues (Including plagiarism, informed consent, misconduct, data fabrication and/or falsification, double publication and/or submission, redundancy, etc.) have been completely observed by the authors.
